# A surface confined yttrium(iii) bis-phthalocyaninato complex: a colourful switch controlled by electrons†
†Electronic supplementary information (ESI) available: Synthetic details and additional characterisation data. See DOI: 10.1039/c6sc00443a


**DOI:** 10.1039/c6sc00443a

**Published:** 2016-04-26

**Authors:** I. Alcón, M. Gonidec, M. R. Ajayakumar, M. Mas-Torrent, J. Veciana

**Affiliations:** a Institut de Ciència de Materials de Barcelona (ICMAB-CSIC) , Networking Research Center on Bioengineering , Biomaterials and Nanomedicine (CIBER-BBN) , Campus de la UAB , 08193 , Bellaterra , Spain . Email: mmas@icmab.es ; Email: vecianaj@icmab.es

## Abstract

SAMs of a Y(iii) double-decker complex on ITO have been prepared and their electrical and optical properties explored, exhibiting three accessible stable redox states with characteristic absorption bands in the visible spectra, corresponding to three complementary colors (*i.e.*, green, blue and red). These absorption bands are exploited as output signals of this robust ternary electrochemical switch, behaving hence as an electrochromic molecular-based device.

## Abstract 2

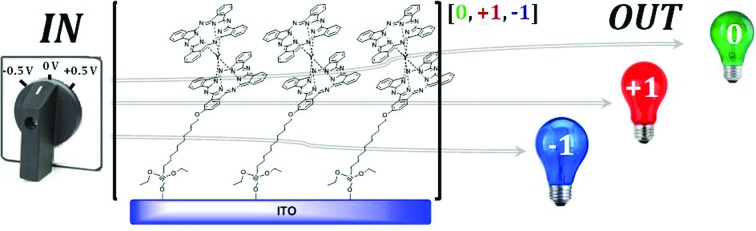
SAMs of a Y(iii) double-decker complex on ITO have been prepared and their electrical and optical properties explored, exhibiting three accessible stable redox states with characteristic absorption bands in the visible spectra, corresponding to three complementary colors (*i.e.*, green, blue and red).

## Introduction

In the last 20 years huge efforts have been devoted to studying and developing organic molecules for electronic applications. Molecules can be, in principle, synthesized in mass production at a relatively low cost and, by chemical design, their properties can be tuned. In order to fabricate devices, molecules are typically supported on inorganic substrates (mainly metals or metal-oxides), facilitating their manipulation and the possibility to direct the application of external stimuli on them. One common route is the fabrication of self-assembled monolayers (SAMs) which is focused on the use of molecules with a specific functional group that spontaneously bonds to the surface.^1^,^2^ Electrochemical molecular switches are a particularly appealing class of molecular devices where electroactive molecules are switched reversibly between different redox states triggered by an electrical signal.^3^ Optical, magnetic, electrical or chemical outputs can be used to read the state of the switch.^4^–^12^ Most of the reported examples are based on bi-stable molecules where the two accessible redox states can be visualized as 1's or 0's mimicking the terminology employed in the binary logic system which is the basis of current memory devices. However, it is known that the fabrication of devices with a higher number of states would facilitate the processing of higher memory densities.^13^,^14^ Despite this interest, only a few examples based on electroactive SAMs that can present three or more states have been reported to date.^13^–^15^ Most of these systems take advantage of the different optical absorption levels that the distinct redox states exhibit at determined wavelengths as the readout mechanism.^16^–^18^ Double and triple-decker phthalocyanine lanthanide complexes are potential building blocks for the fabrication of electrochemical switches owing to their rich electrochemistry that allows an easy access to a range of oxidation states centred on the ligands.^19^–^21^ Lindsey and Bocian demonstrated with Eu phthalocyanine triple-decker SAMs that four available redox states could be accessed electrochemically.^4^ In solution and in thin films it is widely known that the reduction and oxidation processes in these materials are accompanied by significant changes in their optical absorption spectra.^22^–^24^ This prompted us to explore the possibility to exploit this property as the output of a surface confined switch based on a double-decker phthalocyanine lanthanide complex. In this work, a ternary switchable SAM of a bis-phthalocyaninato–Y(iii) complex has been prepared. By the application of a low bias voltage, three redox states have been accessed and clearly identified using optical absorption spectroscopy. Remarkably, each state shows characteristic absorption bands giving complementary colours. The SAMs are revealed to be very robust and stable upon the application of more than 100 switching cycles.

## Results and discussion

### Synthetic procedures

**Scheme 1:**
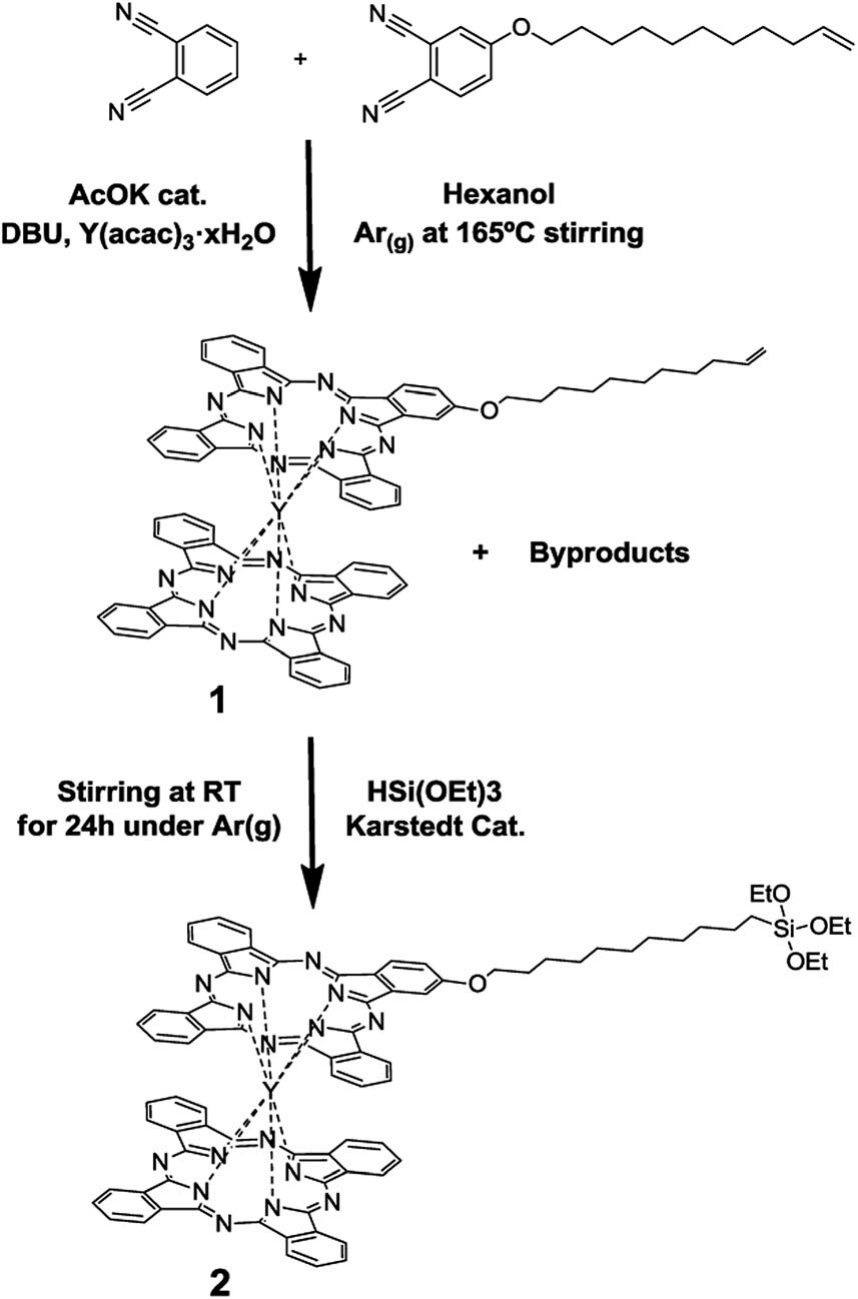
Synthesis of compounds **1** and **2**.

The yttrium(iii) double-decker complex **2** (Scheme 1) was especially designed to form SAMs on indium-tin oxide (ITO). ITO was chosen as the supporting substrate due to its excellent properties for performing spectro-electrochemistry experiments since it is a transparent and electrical conductor oxide. Compound **2** bears a triethoxysilane moiety as a surface anchoring group. Scheme 1 shows the route followed to synthesize it. The first step consisted of the synthesis of compound **1**, a bis-phthalocyaninato yttrium(iii) complex bearing a long alkyl chain with an end-vinyl group, which was carried out in one step adapting methods from the literature.^25^ Firstly, a solution of acetyl-acetonate–Y(iii) (1 eq.), phthalonitrile (6.34 eq.), alkene phthalonitrile (1.6 eq.), a catalytic amount of potassium acetate and diaza-bicycloundecene (DBU, 4 eq.) in hexanol was heated at 165 °C for 14 h. The solid product was extensively purified using a silica-gel chromatography column and a size-exclusion chromatography column for separating **1** from the non-alkylated double-decker complex and the multi-alkylated ones. Compound **1** was obtained in a 12% yield.

Compound **2** was synthesized by dissolving **1** in distilled triethoxy-silane, adding a catalytic amount of Karstedt catalyst (Pt(0)-1,1,3,3-tetramethyldisiloxane 2% solution in xylene) and stirring the solution for 24 h at room temperature. After purification using silica-gel chromatography, compound **2** was obtained in a 35% yield. All the experimental and characterization details of compounds **1** and **2** are provided in the ESI.†

### Electro-optical characterization of **2**

The cyclic voltammetry (CV) of **2** in solution (see ESI†) showed two redox waves at low potential bias corresponding to a one electron reduction process (**2^0^** → **2^–^**) at –0.18 V and to a one electron oxidation process (**2^0^** → **2^+^**) at 0.35 V (*vs.* Ag(s)). Next, UV-vis spectro-electrochemistry experiments were performed to follow the redox inter-conversion between the 3 redox states (**2^+^**, **2^0^** and **2^–^**). A specially designed UV-vis cuvette for performing electrochemistry was used for this purpose with a Pt–Rh net as the working electrode (WE) and Ag and Pt wires as the reference (RE) and counter electrodes (CE), respectively. As an electrolyte, a deoxygenated 50 mM TBAPF_6_ solution in 1,2-dichlorobenzene was employed (TBAPF_6_ = tetrabutylammonium hexafluorophosphate). The electrochemical potentials used to perform the chronoamperometry were –1 V (**2^0^** → **2^–^**), +0.25 V (**2^–^** → **2^0^**), +1 V (**2^0^** → **2^+^**) and –0.1 V (**2^+^** → **2^0^**) *vs.* Ag(s).

**Fig. 1:**
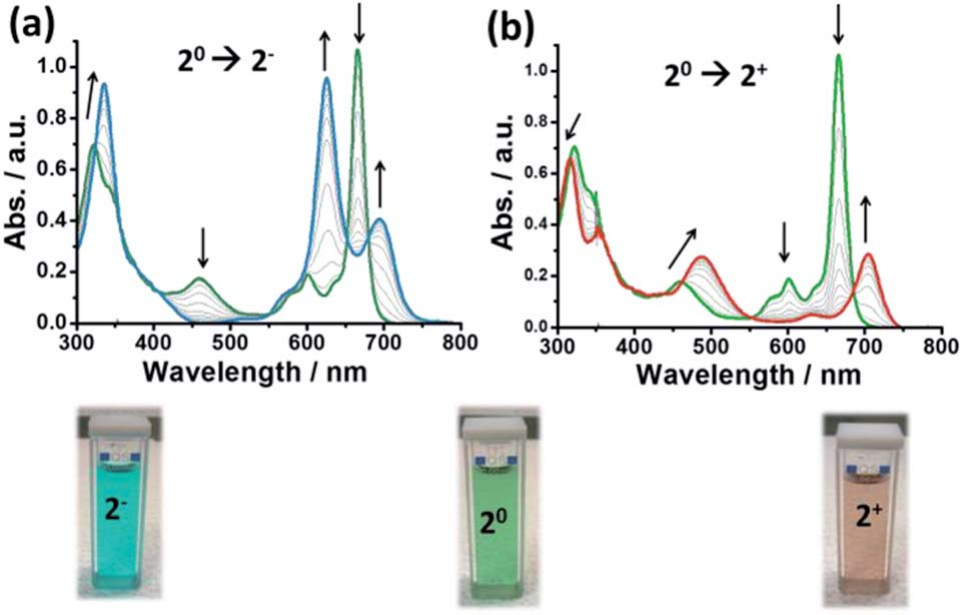
Spectro-electrochemistry experiments for (a) 1e-reduction (**2^0^** → **2^–^**) and (b) 1e-oxidation (**2^0^** → **2^+^**) processes of a solution of 0.1 mM of **2** using a 50 mM TBAPF_6_ solution in 1,2-dichlorobenzene as the electrolyte, a Pt–Rh net as the working electrode and Pt and Ag wires as the counter and reference electrodes, respectively. On the bottom, the solutions of **2** at each redox state are shown.

Fig. 1 illustrates the UV-vis spectra recorded during the experiments where several clear isosbestic points can be observed demonstrating the reversibility of the redox processes. The UV-vis absorption spectrum of the neutral compound showed the typical absorption peaks of this family of compounds: the Soret bands at 334 and 371 nm, the π-radical band at 474 nm, the vibronic bands around 600 nm and the Q-band at 663 nm. Upon reduction, the Soret bands of the neutral complex fuse into a single absorption band at 358 nm. The π-radical band gradually disappears (corroborating the closed-shell nature of the reduced species) and the neutral Q-band at 663 nm splits into two bands (625 and 690 nm). Upon oxidation, the Soret band is blue-shifted. Also, the π-radical band at 474 nm shifts to higher wavelengths and increases in intensity (in accordance with the bi-radical character of the oxidized species). The vibronic bands of the neutral state (600 nm) disappear and, finally, the Q-band at 663 nm moves to lower energies and decreases in intensity. Remarkably, with the naked eye one can observe that the green solution of **2** (*c* = 0.1 mM) becomes blue upon reduction and red when it is oxidized (see Fig. 1, below). Such colour complementarity between the different redox states in solution is highly appealing for its exploitation in electro-optical molecular switches.

### **2**-SAM preparation

**Fig. 2:**
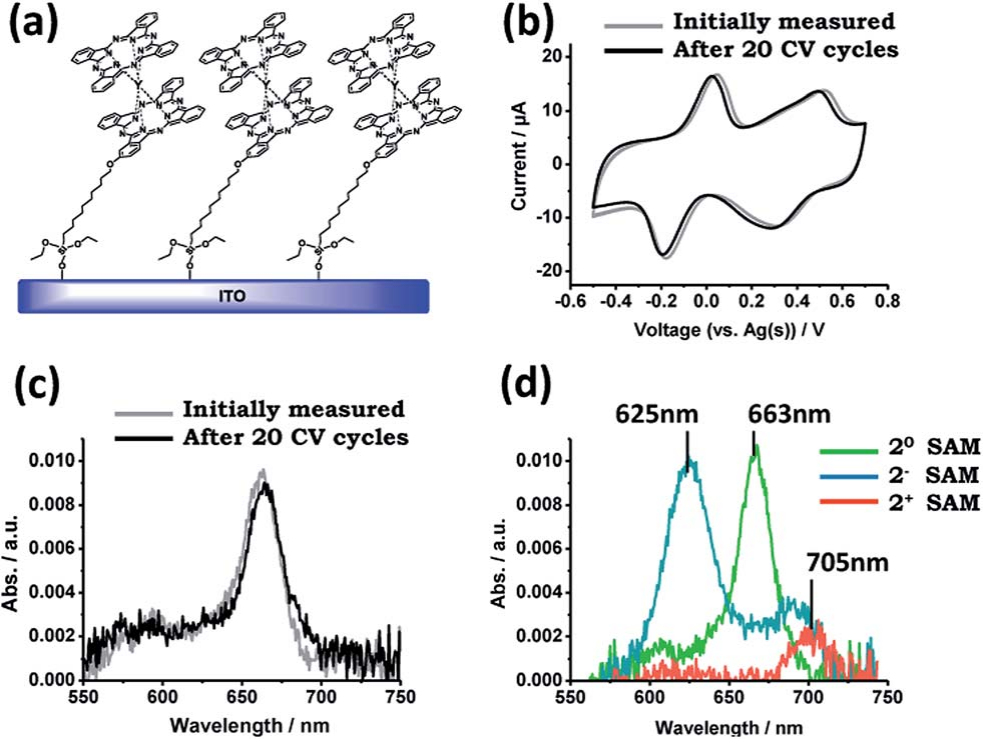
(a) Scheme of **2**-SAM. (b) CV of **2**-SAM used as the WE and Pt and Ag wires as the CE and RE, respectively, in a 50 mM TBAPF_6_ solution in 1,2-dichlorobenzene at a scan rate of 0.3 V s^–1^. (c) Vis absorption spectra of **2**-SAM. The grey and black lines in (b) and (c) are the results before and after applying 20 CV cycles, respectively. (d) Vis absorption spectra of **2**-SAM in the neutral, **2^0^**, oxidised, **2^+^**, and reduced, **2^–^**, states.

Once the excellent electro-optical properties of **2** for the purpose of this work had been corroborated, the following step was the anchoring of the active molecule on a solid support, in this case ITO. The ITO double coated-glass substrates were chemically activated by being immersed into an oxidant solution NH_4_OH : H_2_O_2_ : H_2_O (1 : 1 : 5), rinsed with water and acetone, and dried with a N_2_ flow. Immediately, the substrates were immersed in a 0.4 mM solution of **2** in toluene. The solution was heated at 75 °C for 1 hour and then maintained at 45 °C for 14 hours under an argon atmosphere. After that time the ITO slides were removed from the organic solution, rinsed with toluene to remove the physisorbed material and dried with a N_2_ stream to give the **2**-SAM (Fig. 2a). X-ray photoelectron spectroscopy (XPS) and time-of-flight secondary ion mass spectrometry (ToF-SIMS) characterization was carried out confirming the formation of the monolayer (ESI†).

### Electro-optical characterization of **2**-SAM

Besides the elemental surface characterization, the electrochemical and optical properties of **2**-SAM were also studied. CV experiments were performed using the functionalized substrate as the WE and Ag and Pt wires as the RE and CE, respectively, in a 50 mM TBAPF_6_ solution in 1,2-dichlorobenzene as the electrolyte medium. As can be seen in Fig. 2b, two redox processes appeared in the CV at similar potential values as previously observed for this molecule in solution: the reduction process (**2^0^**-SAM → **2^–^**-SAM) appeared at –0.07 V (*vs.* Ag(s)) and the oxidation process (**2^0^**-SAM → **2^+^**-SAM) at +0.42 V (*vs.* Ag(s)). These results demonstrate that the electro-active nature of **2** is preserved after being surface bonded. It is worth noting that the wide peak observed in the CV for the oxidation of **2**-SAM could be caused by the presence of molecules with different chemical environments.^26^ The current intensity of both waves linearly increased with the applied scan rates (*i.e.*, 0.05, 0.1, 0.2, 0.3 and 0.4 V s^–1^), which is characteristic of surface-confined electroactive species (ESI†). Further, for testing the robustness of the **2**-SAM upon electrical stress, 20 cycles between –0.7 and +0.8 V were applied without observing any loss in the current intensity (Fig. 2b).

Visible absorption characterization was also performed for **2**-SAM before and after applying the 20 CV cycles (Fig. 2c). Since it is known that the ITO functionalized substrates exhibit a broad band in the region of 400–500 nm,^2^ we focused on the absorption characteristics above this region. In the spectrum, the typical Q-band absorption (*λ* = 663 nm) corresponding to the neutral state is observed. The initial recorded spectrum perfectly matches the one measured after the application of 20 CV cycles, pointing out, again, the good stability of the **2**-SAM.

### Switch-ability of **2**-SAM

The next step was to explore the possibility to employ the optical properties to follow the electrochemically triggered changes in **2**-SAM. For this, the absorption spectra were registered in the 550–750 nm range while sequentially applying pulses of different voltages over 2 minutes for generating each accessible redox state in the **2**-SAM (*i.e.*, +0 V for **2^0^**-SAM, –0.4 V for **2^–^**-SAM and +0.6 V for **2^+^**-SAM). In Fig. 2d the absorption spectra of the three redox states of **2**-SAM are shown, observing clear fingerprints for each of them. Indeed, the maximum absorption bands at 625, 663 and 705 nm can be employed to identify unambiguously the states **2^–^**-SAM, **2^0^**-SAM, and **2^+^**-SAM, respectively. That is, these absorption bands can be used as output signals.

**Fig. 3:**
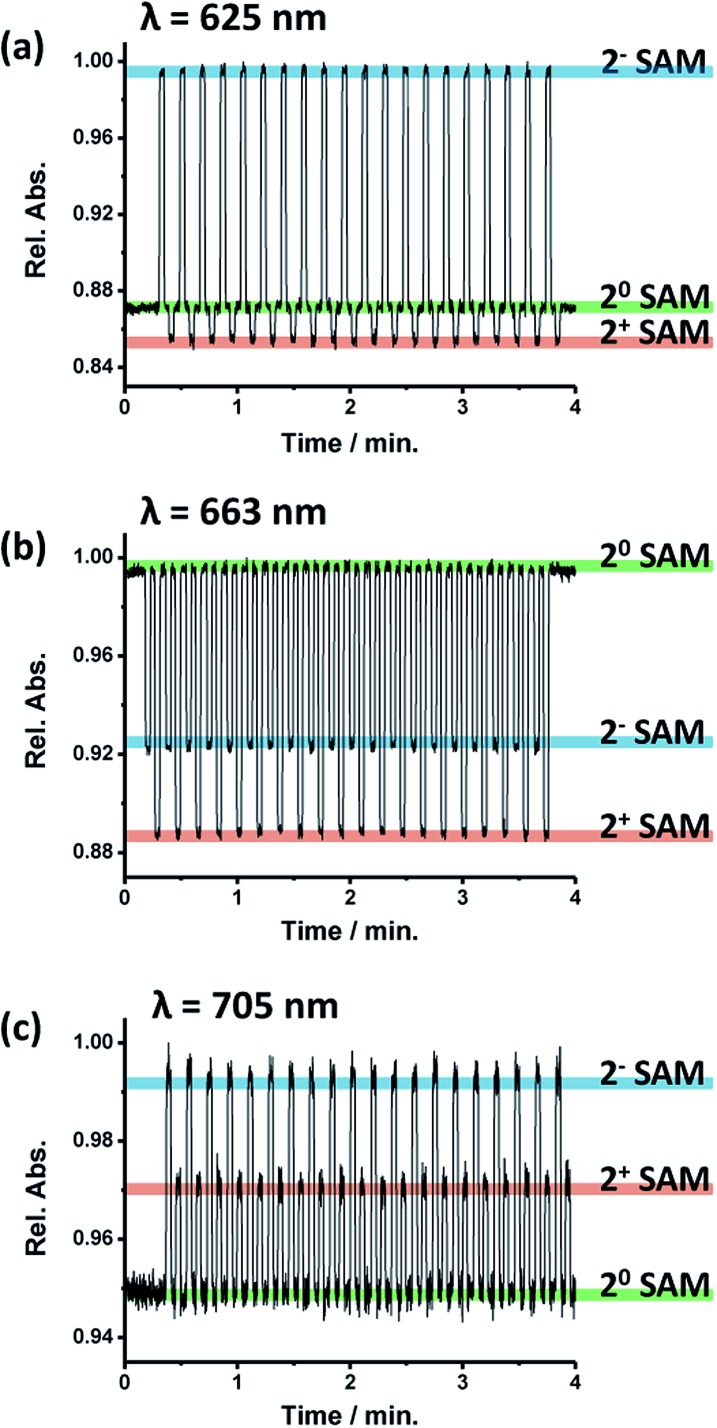
Application of 20 electrochemical switching cycles to the **2**-SAM while measuring the absorbance at each of the three characteristic wavelengths: (a) 625 nm, (b) 663 nm and (c) 705 nm. Each potential pulse was applied for 3 s. The different absorption plateaus correspond to the different states of the switch, highlighted with the corresponding colours.

The evolution of the absorption of **2**-SAM at the three characteristic wavelengths of the maximum absorption bands (*i.e.*, 625, 663 and 705 nm) was investigated when switching the surface between the different states by applying the corresponding voltage value for a 3 s pulse (Fig. 3). It was observed that the three wavelengths can be exploited to track the state of the switch since at each wavelength **2^0^**-SAM, **2^–^**-SAM and **2^+^**-SAM exhibit different levels of absorption. However, a wavelength of 663 nm seems to be more suitable since the three states have more separated absorption intensities. Table 1 summarizes the **2**-SAM switching behaviour, where high absorption values are labelled as “H”, medium as “M” and low as “L”. This molecular device was switched between the three states for more than 100 cycles without signal loss, unambiguously demonstrating the robustness of the system.

**Table 1:** Truth table for the **2**-SAM switch

Input (V *vs.* Ag(s))	Logic state	Redox state	Output^*a*^ I (625 nm)	Output^*a*^ II (663 nm)	Output^*a*^ III (705 nm)
0	0	**2^0^**-SAM	M	H	L
–0.4	1	**2^–^**-SAM-	H	M	H
+0.6	2	**2^+^**-SAM	L	L	M

^*a*^The letters L, M and H in the table body indicate low, medium and high absorption intensity levels, respectively.

We have found that the time required for the complete switching between the different redox states of the **2**-SAM is 0.3–0.4 s (see ESI†). This value is in accordance with other reported electro-active SAMs on ITO.^5^

### Stability of the **2**-SAM switch states

**Fig. 4:**
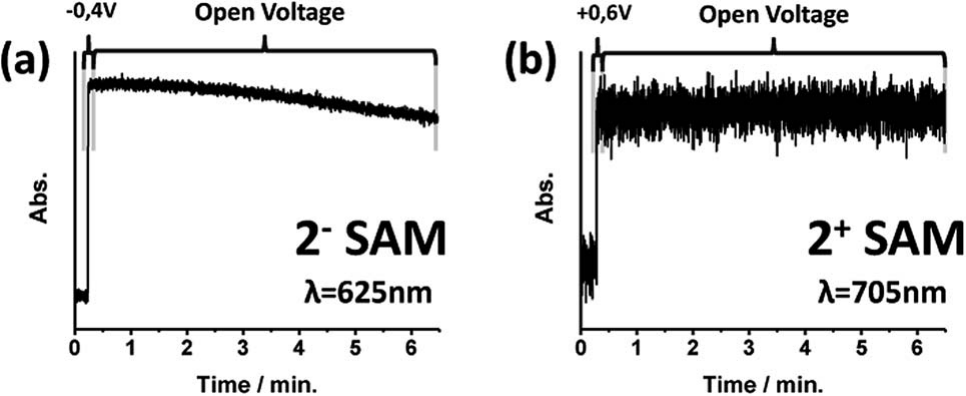
Stability of the electro-generated **2^–^**-SAM and **2^+^**-SAM states followed by the UV-vis absorption of the surface at 625 and 705 nm, respectively. Potential pulses of 10 s were applied before leaving the system at open voltage.

The preservation of oxidized and reduced states without the application of an external stimulus was also tested. Voltage pulses were applied to electro-generate the corresponding redox state and then the system was left at open voltage. Each state was followed by recording the absorption of its characteristic band (Fig. 4). After 2 minutes, the reduced **2^–^**-SAM state started to show a decrease in its absorption band, while the oxidized **2^+^**-SAM did not reveal any sign of deterioration for more than 7 minutes. The lower stability of the **2^–^**-SAM state compared with **2^0^**-SAM and **2^+^**-SAM is in accordance with the behaviour of reduced double-decker derivatives in solution.^27^

Finally, the shelf-stability of the **2**-SAM was also explored when the functionalized substrate was kept at room temperature and in environmental conditions. Using absorption spectroscopy, it was demonstrated that after one month and a half there was no signal loss (ESI†).

## Conclusions

In summary, a novel phthalocyanine double-decker Y(iii) complex has been designed and synthesized to be grafted on oxide substrates. SAMs of this molecule on ITO, a conducting and transparent substrate, have been prepared and their electrochemical and optical properties explored. This system exhibits three accessible redox states at relatively low voltages and behaves as a stable ternary electrochemical switch. Outstandingly, each state reveals clear characteristic absorption bands in the visible spectra, which are in fact responsible for the three complementary colours that the solutions of each redox state of this compound show (*i.e.*, green, blue and red). Such absorption bands in the SAM were exploited as output signals of the system, behaving hence as an electrochromic molecular-based device. The high robustness and stability that the system revealed point towards the high potential that phthalocyanine lanthanide complexes have as molecular electro-optical switches.

## Supplementary Material

Supplementary information
