# Supplementary material for: A surface confined yttrium(iii) bis-phthalocyaninato complex: a colourful switch controlled by electrons
Source: Chem Sci. 2016 Apr 26;7(8):4940–4. doi: 10.1039/c6sc00443a (PMC6018459; doi:10.1039/c6sc00443a)
Supplement: Supplementary file 1 [file SC-007-C6SC00443A-s001.pdf]

Electronic Supplementary Material (ESI) for Chemical Science.
This journal is © The Royal Society of Chemistry 2016

SUPPORTING INFORMATION

A SURFACE CONFINED YTTRIUM(III) BIS-PHTHALOCYANINATO COMPLEX: A COLOURFUL SWITCH CONTROLLED BY ELECTRONS

Isaac Alcon, Mathieu Gonidec, M. R. Ajayakumar, Marta Mas-Torrent*, Jaume Veciana*

TABLE OF CONTENTS

**Synthesis of Compound 1**	S2
**Synthesis of Compound 2**	S5
**Preparation of 2-SAM on ITO**	S7
**Characterization of the 2-SAM**	S8-S11
**X-ray Photo-electron Spectroscopy (XPS)**	S8
**Time-of-Flight Secondary Ion Mass Spectrometry (ToF-SIMS)**	S9
**Cyclic Voltammetry (CV)**	S10
**Time response of 2-SAM switch**	S11
**Stability of 2-SAM on ITO over time**	S11

SYNTHESIS OF COMPOUND 1

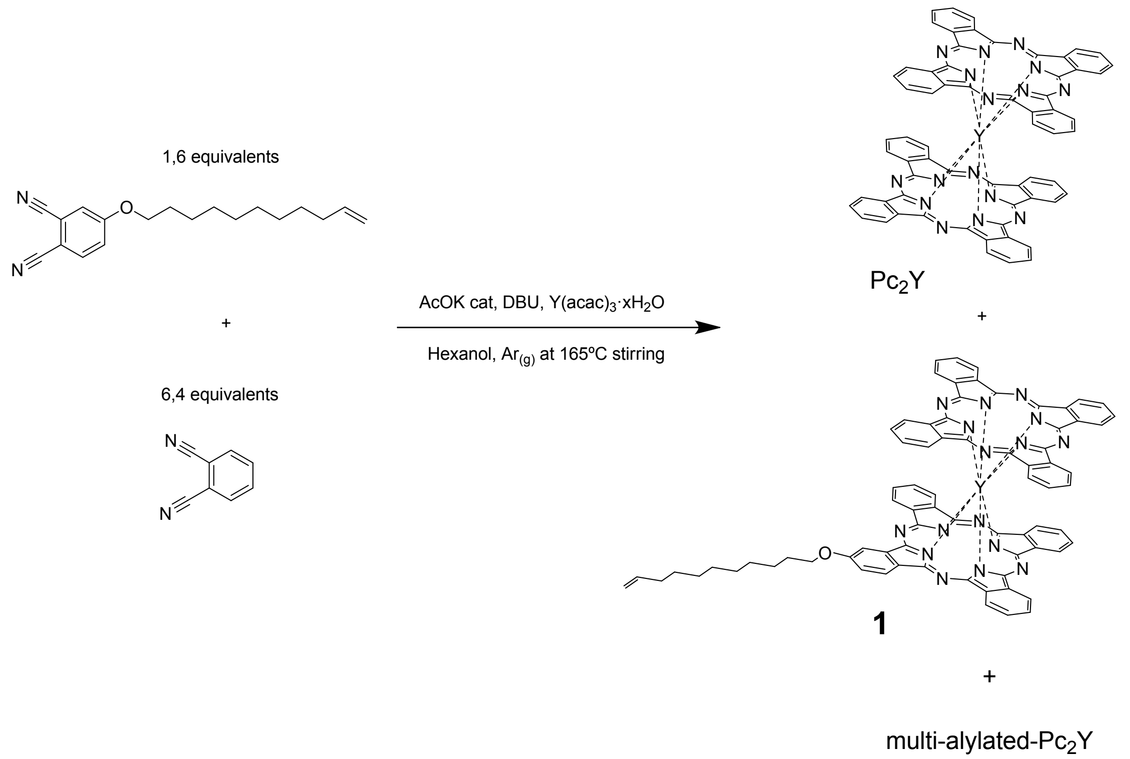

In a flame dried Schlenk tube 567 mg of phthalonitrile (4.43 mmol 6.34 eq.), 282 mg of acetyl-acetonate yttrium(III) (0.698mmols 1eq), 9 mg of potassium acetate (0.1 mmol catalytic amount) and 328 mg of 1,2-Benzenedicarbonitrile-4-(10-undecen-1-yloxy) (1.12mmol 1.6 eq.) were carefully added. Then 10 mL of hexanol were added and the mixture was stirred for 5 min at room-temperature. After that time 420 µL of diaza-bicycloundecene (DBU; strong organic base) were introduced and the solution was stirred at 165°C for around 14 h.

Then the solvent was removed by an air flux keeping the mixture at 165°C. The green solid was dissolved in 500 mL of dichloromethane and several volumes of acetic acid (50 mL) were added until the solution turned from blue to a greenish color. Several extractions with distilled water were done to remove DBU and other ionic and highly polar species form the organic medium. The organic fractions were dried with generous amounts of magnesium sulfate while stirring the solution. The inorganic solid was removed by filtration, the solvent was evaporated in a rotary evaporator and the remaining green precipitate was suspended in methanol. The suspension was filtered in vacuum through a NYLON filter. The green precipitate was dissolved in dichloromethane and eluted through a SiO_2-gel column. The 2nd fraction of this column (green color) was passed through a size-exclusion chromatography column a couple of times until the mono-alkylated-DD(Y) complex (**1**) was completely separated from the firstly eluted non-alkylated complex (Pc_2Y) and the multi-alkylated double-decker species, which corresponded to the later eluted fractions. Compound **1** was characterized by MALDI-TOF mass spectroscopy, thin layer chromatography, UV-Vis and IR-ATR spectroscopy and cyclic voltammetry. The final green compound **1** was obtained in a 12% yield (36 mg).

Rf_1 (*SiO_2 TLC, eluent = $CHCl_3$*) = 0.49

UV-Vis (log(ε)): 663 (5.078), 633 (4.246), 600 (4.332), 576 (4.137), 457 (4.310), 343 (4.750), 320 (4.916) nm.

CV: $E^{1/2}_{(0\leftrightarrow+1)}$ 0.266 V, $E^{1/2}_{(0\leftrightarrow-1)}$ -0.221 V

IR-ATR(cm^{-1}): 679, 697, 720, 737, 773, 810, 882, 1000, 1058, 1158

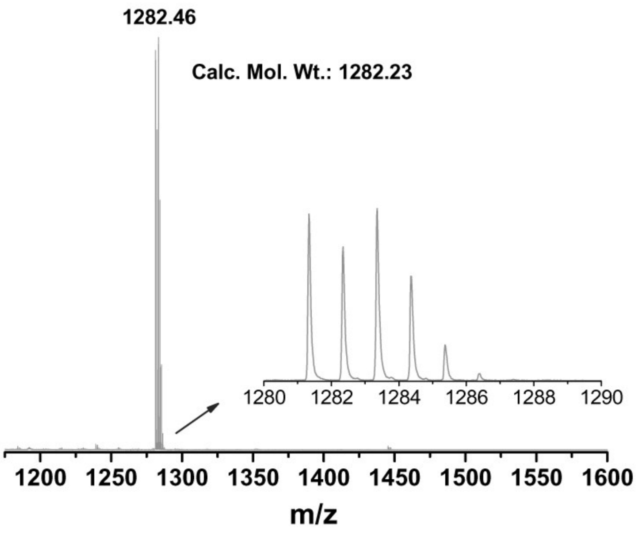

Figure S1. MALDI-ToF mass spectrum of **1** at negative mode.

ESR spectrum in CH_2Cl_2 at 298 K: 1 signal at g = 2.00172.

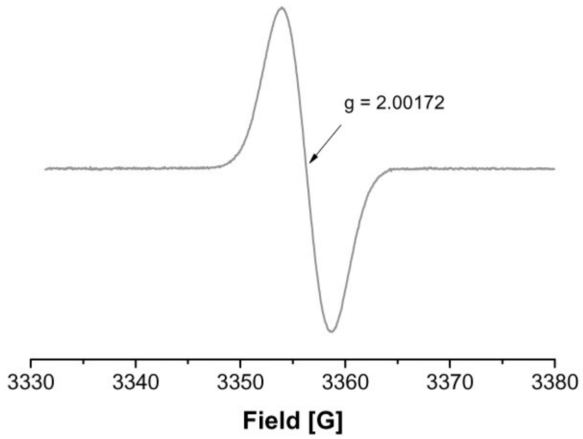

Figure S2. ESR spectrum of **1** in CH_2Cl_2 at 298 K.

Nuclear Magnetic Resonance (NMR) spectroscopy of Compound 1: ^{1}H NMR spectra were recorded on a Bruker 400 MHz spectrometer in CD2Cl2 with TMS as a standard. Spin multiplicities are reported as singlet (s), and doublet (d), with coupling constants (J) given in Hz, or multiplet (m). The aromatic protons (α, α′, α′′, β and β′) of compound **1** are NMR silent due to the paramagnetic nature of the molecule, however, its vinylene and aliphatic protons gave respective signals.

^{1}H NMR (400 MHz, CD_2Cl_2): δ = 5.99-5.87 (m, 1 H; —CH=), 5.14-4.98 (m, 2 H; CH_2=), 2.25-1.35 (m, alkylCH_2).

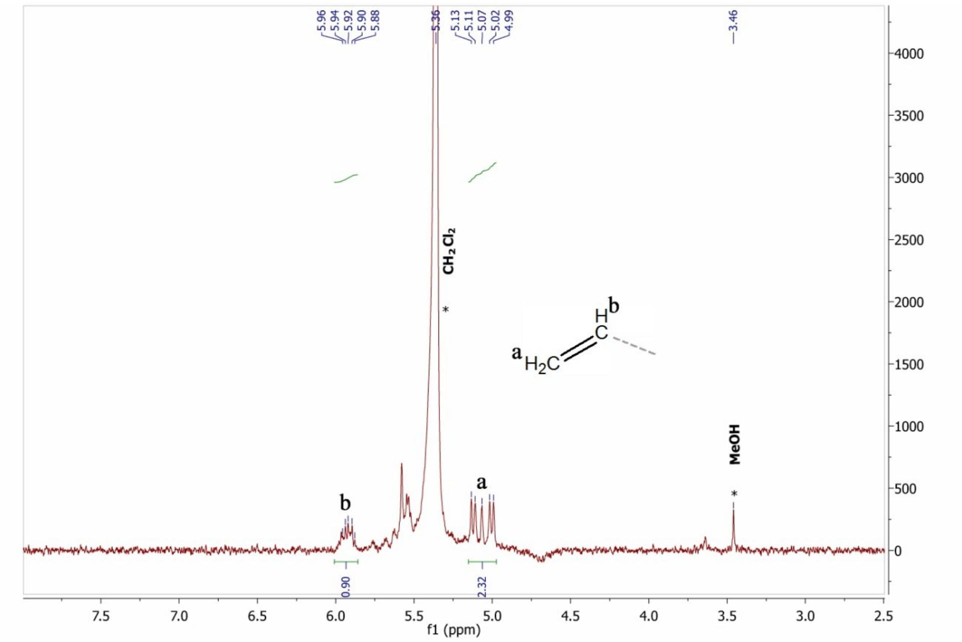

Figure S3. ¹H NMR spectra of **1** in CD_2Cl_2. * denotes the solvent impurity.

On treatment with hydrazine hydrate (NH_2-NH_2 . xH_2O), as a reducing agent, compound **1** gave well-resolved ¹H NMR spectra. The disappearance of vinylene protons could be due to a possible reaction with NH_2-NH_2.[1-3]

¹H NMR (400 MHz, CD_2Cl_2/NH_2-NH_2 .xH_2O): δ = 8.99 (d, J = 3.2, 14 H; H-α), 8.87 (d, J = 8.3, 1 H; H-α′′), 8.47 (s, 1 H; H-α′), 8.20 (s, 14 H; H-β), 7.79 (d, J = 8.1, 1 H; H-β′), 2.35-1.34 (m, 18 H; alkylCH$_2$).

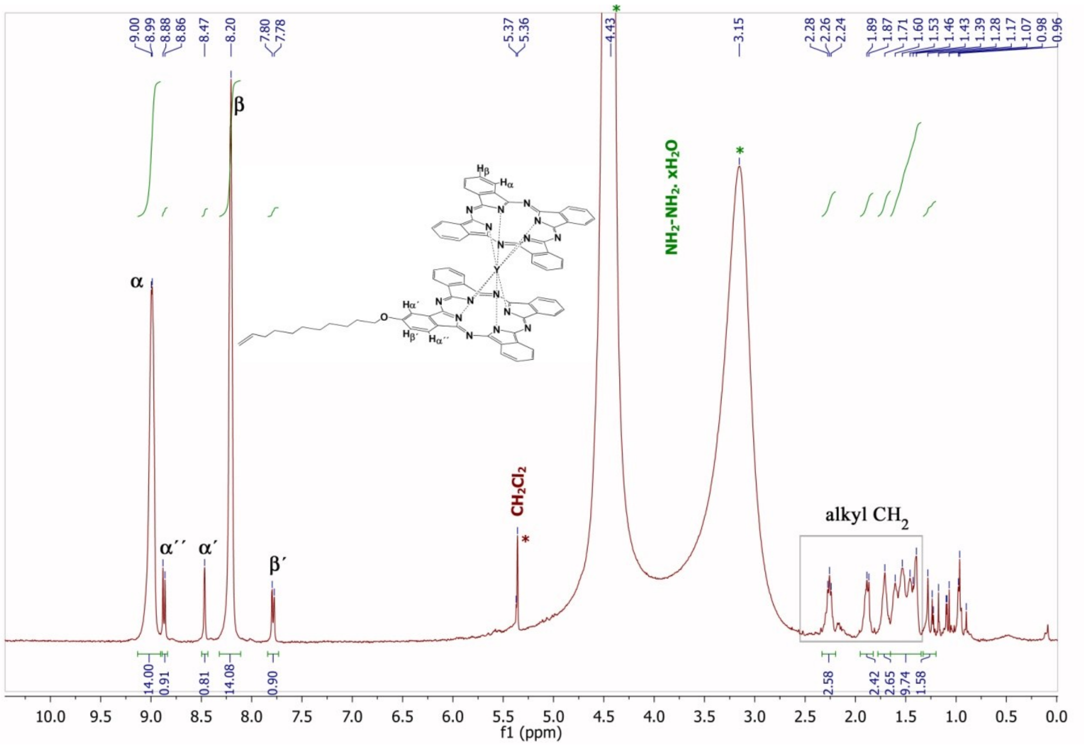

Figure S5. ¹H NMR spectra of **1** recorded in CD_2Cl_2 in presence of NH_2-NH_2.xH_2O. * denotes the solvent impurity.

SYNTHESIS OF COMPOUND 2

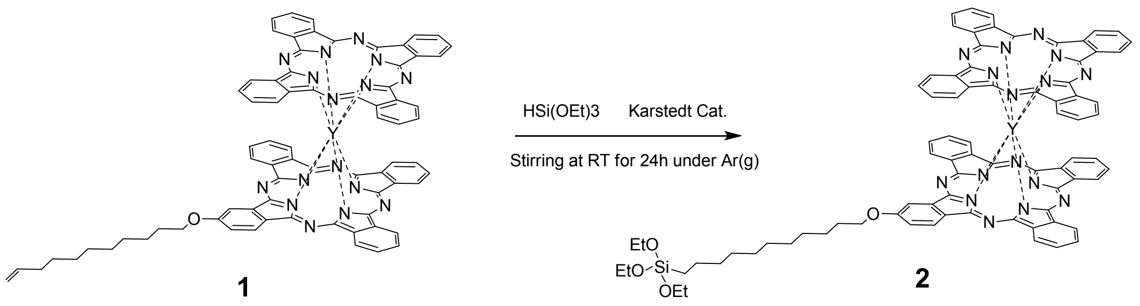

In a flame dried Schlenck under argon, 36 mg of compound **1** (28 µmol, 1.0 eq.) were dissolved in 0.4 mL of triethoxysilane. 50µl of a 2% solution of Karstedt catalyst (Pt(0)-1,1,3,3-tetramethyldisiloxane complex) in xylene was added and the mixture was stirred at room temperature under argon for 24 h. The reaction progress was followed by thin layer chromatography (SiO_2 on aluminum foil, $CHCl_3$ as eluent). After around 24h the solvent $HSi(OEt)_3$ was removed by distillation in vacuum at 72°C. The green solid was dissolved in some milliliters of chloroform and eluted through a silica-gel chromatography column using chloroform as eluent. The second green collected fractions corresponded to compound **2**. Once evaporated the solvent in the rotary evaporator in vacuum, 14 mg of the title compound (yield = 35%) were obtained and kept under Ar(g) atmosphere to avoid polymerization caused by atmospheric water. Compound **2** was characterized by MALDI-TOF mass spectroscopy, UV-vis and IR-ATR spectroscopies, thin layer chromatography and cyclic voltammetry.

Rf_2 (*SiO_2 TLC, eluent = $CHCl_3$*) = 0.30

IR(cm-1): 680, 724, 774, 810, 884, 958, 1164, 1538

UV-Vis (log(ε)): 663 (5.044), 633 (4.246), 600 (4.328), 576 (4.132), 457 (4.303), 343 (4.746), 320 (4.912) nm.

CV (*vs* $Ag_{(s)}$): $E^{1/2}_{(0\leftrightarrow+1)}$: 0.37 V; $E^{1/2}_{(0\leftrightarrow-1)}$: -0.18 V

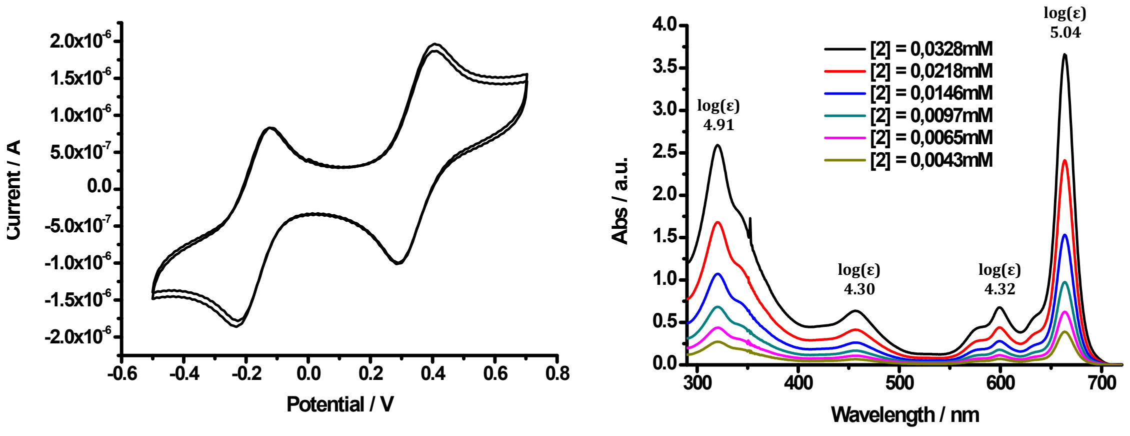

(a) **(b)**

Figure S6. (a) Cyclic Voltammetry of compound **2.** The CV was performed in a 50 mM $TBAPF_6$ solution in 1,2-Dichlorobenzene at a scan rate of 0.1 V/s. Pt wires were used as WE and CE and a Ag one was used as RE. (b) UV-Vis spectra of compound **2** at different concentrations in HPLC toluene.

MALDI-ToF Mass Spectroscopy (MW: 1446.5 g/mol)

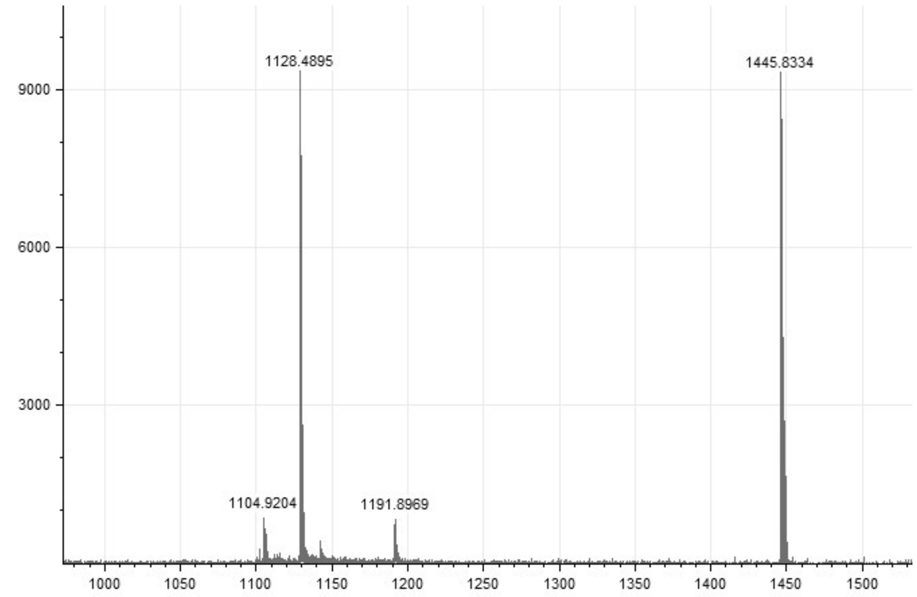

Figure S7. MALDI-TOF mass spectrum of **2** at negative mode.

PREPARATION OF 2-SAM ON ITO

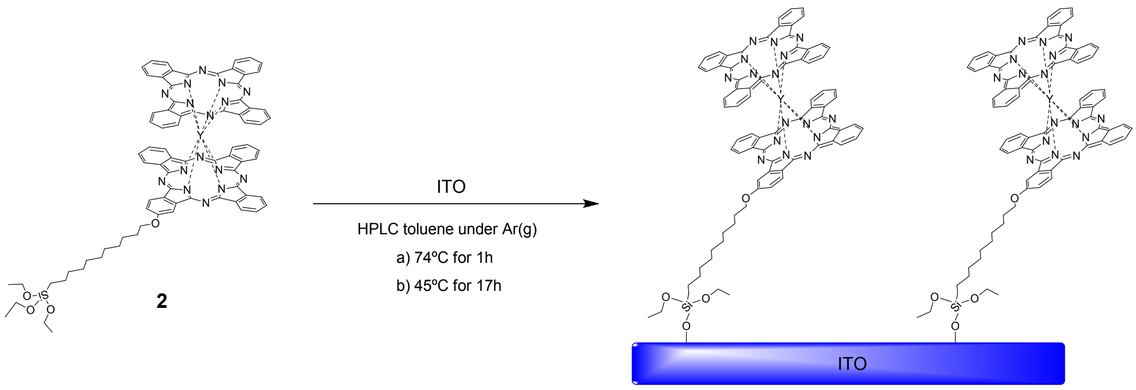

2-SAM

Material Preparation

All the material employed for the SAM preparation was rinsed with DCM, acetone (both HPLC grade), distilled water and MilliQ water. Afterwards the material was placed in a plastic bucket filled with a 1%Hellmanex solution in MilliQ water for 3h. The bucket was emptied, filled with MilliQ water and left for 30 min. This was repeated twice. The material was removed, generously rinsed with MilliQ water and placed inside the oven at 60°C overnight.

Degreasing ITO slides

The ITO slides were sonicated in HPLC DCM for 15 min. Then the slides were rinsed with the same solvent and dried with a $N_{2(g)}$ stream. The entire process was repeated with HPLC acetone and ethanol.

Activation of ITO and **2**-SAM formation

The ITO slides were activated in an oxidant bath (MilliQ-water:$H_2O_2^{(50\%)}$:$NH_4OH^{(5N)}$; 5:1:1) at 68°C – 75°C for 35 min.

The solution was degassed for 10 min more with an $Ar_{(g)}$ stream and heated between 73 – 75°C for 1h in Ar atmosphere. After that time the temperature was decreased to 45°C and the solution was left at that temperature under inert conditions ($Ar_{(g)}$ atmosphere) overnight. Next, the ITO slides were taken out of the solution, rinsed with HPLC toluene and dried with a $N_{2(g)}$ stream.

Meanwhile, a solution of 1.7 mg of compound **2** in 3 mL of HPLC toluene was prepared. The solution was sonicated for 2 min and then was filtered through a 0.2 µm-filter. Then, the solution was degassed by bubbling $Ar_{(g)}$ for 10 min.

The ITO slides were taken out of the oxidant solution, rinsed with MilliQ water and HPLC acetone, dried with a $N_{2(g)}$ stream and introduced rapidly into the toluene solution of **2**.

CHARACTERIZATION OF THE 2-SAM

X-Ray Photo-electron Spectroscopy (XPS)

Element	Beam Energy Peak/eV
In3d	**444.45**
Sn3d	**486.78**
O1s	**530.53**
C1s	**285.25**
N1s	**398.5**
Si2p	**102.15**
Y3d	**158.36**

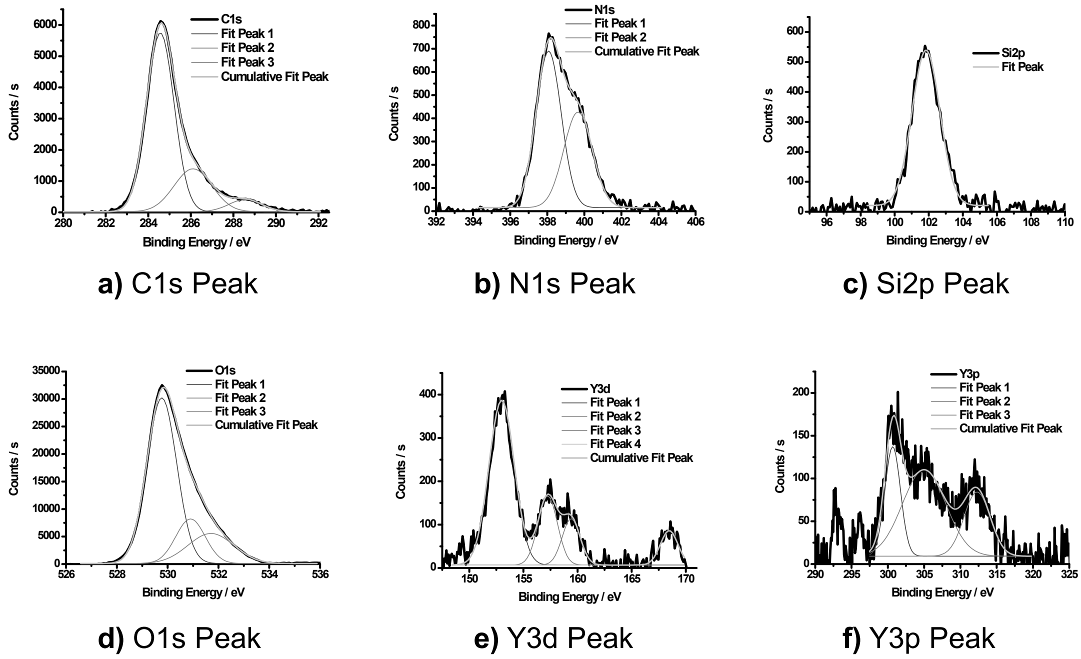

Figure S8. XPS peaks with the corresponding deconvolution analysis of C1s, N1s, Si2p, O1s and Y3d and 3p (a, b, c, d, e and f, respectively). Due to a possible overlap between the Y3d peak and the Si2s one, the energy of the Y3p orbital was also scanned.

Time-of-Flight Secondary Ion Mass Spectrometry

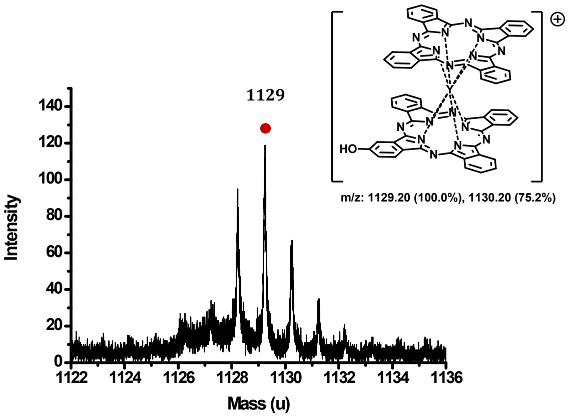

Figure S9. Zoomed ToF-SIMS spectrum of the 2-SAM. The peak at m/z = 1129 corresponds to the fragment depicted on the top-right side. This peak was also detected in MALDI-TOF mass spectroscopy measurements of compounds 1 and 2.

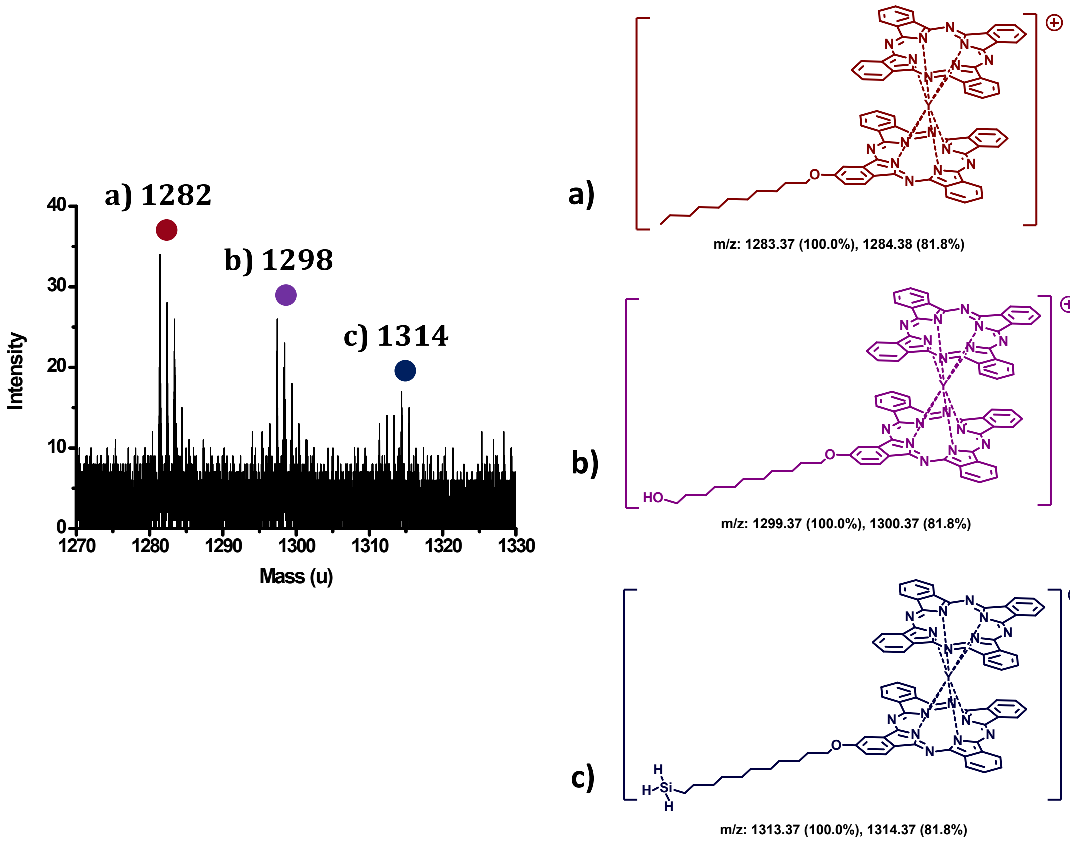

Figure S10. Zoomed ToF-SIMS spectrum of the 2-SAM. The three highlighted peaks correspond to the three ionic structures showed in the right side (a, b and c).

Cyclic Voltammetry

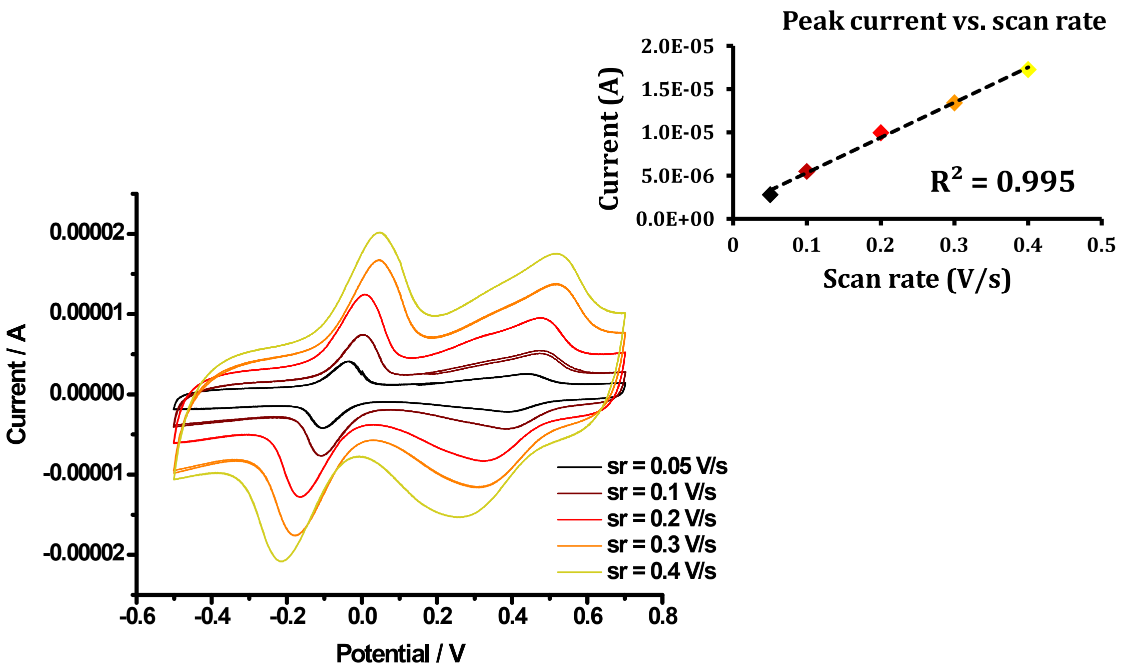

Figure S11. ***Left;*** 2-SAM cyclic voltammetry measurements performed at different scan rates. ***Top-right;*** Reduction peak current (y-axis) against scan rate used (x-axis). The 2-SAM functionalized ITO was used as WE, whereas Ag and Pt wires were used as reference and counter electrodes, respectively. A 50mM $TBAPF_6$ solution in 1,2-Dichlorobenzene was utilized as electrolyte.

TIME RESPONSE OF THE 2-SAM SWITCH

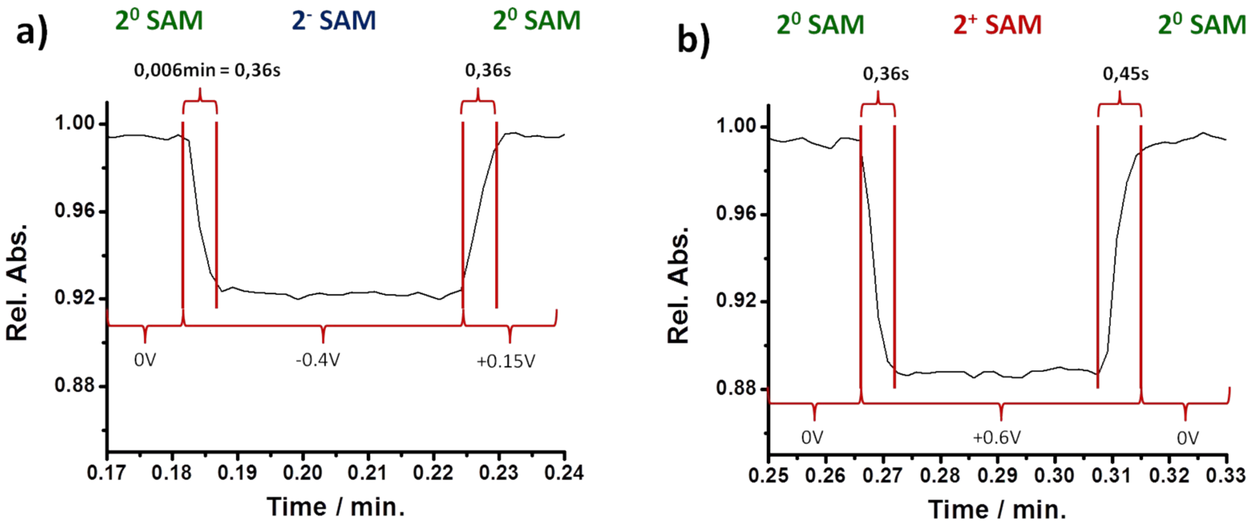

Figure S12. Time-response of the **2**-SAM when electro-generating the 1e-reduced (a) and 1e-oxidized (b) states of the switch by applying the corresponding indicated potential pulses. In this case, the optical output was followed by the absorbance at λ = 663 nm.

STABILITY OF 2-SAM ON ITO OVER TIME

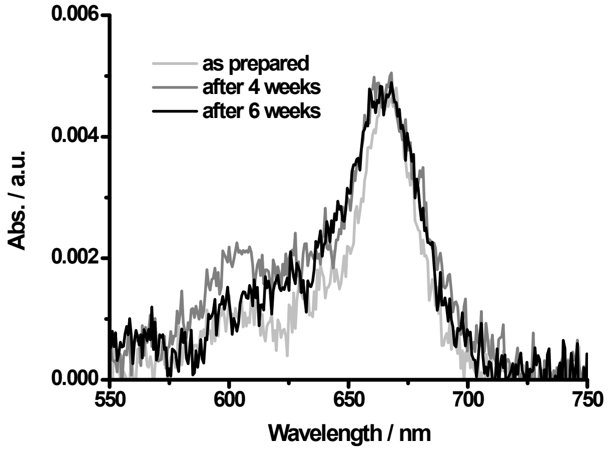

Figure S13. **2**-SAM UV-Vis spectra measured during one month and a half. The **2**-SAM was kept at room temperature and air during that time.

References

[1] M. Ahmed, R. Jackstell, A. M. Seayad, H. Klein and M. Beller, *Tetrahedron Lett.*, **2004**, *45*, 869–873.

[2] Y. Imada, H. Iida, and T. Naot, *J. Am. Chem. Soc.*, **2005**, *127*, 14544-14545.

[3] Y. Imada, T. Kitagawa, T. Ohno, H. Iida, and T. Naota, *Org. Lett.*, **2010**, *12*, 32-35.
